# Age-Related Changes in Serum Lipid Levels, Hepatic Morphology, Antioxidant Status, Lipid Metabolism Related Gene Expression and Enzyme Activities of Domestic Pigeon Squabs (*Columba livia*)

**DOI:** 10.3390/ani10071121

**Published:** 2020-07-01

**Authors:** Qianqian Xu, Huaiyu Li, Wenting Zhou, Xiaoting Zou, Xinyang Dong

**Affiliations:** Key Laboratory for Molecular Animal Nutrition of Ministry of Education, College of Animal Sciences, Zhejiang University (Zijingang Campus), Hangzhou 310058, China; qianqianxu@zju.edu.cn (Q.X.); 21817082@zju.edu.cn (H.L.); 21817022@zju.edu.cn (W.Z.); xtzou@zju.edu.cn (X.Z.)

**Keywords:** pigeon, antioxidant status, lipid metabolism, age-related

## Abstract

**Simple Summary:**

Research on antioxidant status and lipid metabolism in pigeon squabs as meat type poultry is limited. The objective of this study was to explore the age-related changes in the antioxidant status and lipid metabolism of pigeon squabs (*Columba livia*). Ten squabs were randomly selected, out of 400 hatched squabs, on the following days: day of hatching (DOH) and days 7 (D7), 14 (D14), 21 (D21) post-hatch, respectively. BW, serum lipid levels, antioxidant capacity indices, lipid metabolism-related enzyme activities, lipid metabolism-related gene expression, and liver morphology were determined. Results indicated that the phase from DOH to D14 was a growth spurt, especially in the first seven days. The antioxidant capacity of squabs had a continuous decline from DOH to D14. Besides, changes regarding lipid metabolism mainly occurred in the phase from DOH to D14. The first seven days mainly showed less lipid breakdown, while the second seven days displayed more complicated lipid metabolism. The results obtained from this study suggested that, in the pigeon industry, it is better to take nutritional manipulation in squabs before D14.

**Abstract:**

The objective of this study was to evaluate the age-related changes in antioxidant status and the lipid metabolism of pigeon squabs (*Columba livia*), by determining the BW, antioxidant indices, serum lipid levels, lipid metabolism-related enzyme activities, lipid metabolism-related gene expression, and liver morphology in squabs. Ten squabs were randomly selected and sampled on the day of hatching (DOH), days 7 (D7), 14 (D14) and 21 (D21) post-hatch, respectively. The results showed that BW of squabs increased linearly from DOH to D21. The minimum fold of BW gain was observed in the phase from D14 to D21. Serum triglyceride and free fatty acid levels displayed linear and quadratic trends as age increased, with these maximum responses in D14. Serum low-density lipoprotein cholesterol level responded to age linearly and quadratically with the minimum in D14. Serum high-density lipoprotein cholesterol level and the ratio of high-density lipoprotein cholesterol to low-density lipoprotein cholesterol increased linearly with age, whereas the very low-density lipoprotein cholesterol level decreased linearly. The activities of glutathione peroxidase, catalase, and superoxide dismutase in liver displayed linear and quadratic trends as age increased, with these minimum responses in D14. Hepatic malondialdehyde concentration responded to age linearly and quadratically, with the maximum in D14. Activities of lipoprotein lipase, hepatic lipase, and 3-hydroxy-3-methyl glutaryl coenzyme A reductase in liver responded to age linearly and quadratically, with these minimum responses in D14. Hepatic hormone-sensitive lipase activity displayed linear and quadratic trends as age increased with the maximum in D14. Hepatic acetyl CoA carboxylase activity on D14 was significantly lower than squabs on DOH and D7. Hepatic carnitine palmitoyltransferase 1 mRNA expression responded to age linearly and quadratically, with minimum response in D14. Hepatic mRNA expression of acetyl CoA carboxylase and fatty acid synthetase increased linearly with age. Hepatic Oil-Red-O staining area displayed a quadratic trend as age increased, with the maximum response in D14. In conclusion, the phase from DOH to D14 was a crucial development stage for growth, antioxidant status and lipid metabolism in pigeon squabs. The results suggest it is better to take nutritional manipulation in squabs before D14.

## 1. Introduction

As an old Chinese saying goes, “a pigeon is better than nine chickens, and no pigeon can’t make a banquet”. Well known for its flavorful nutritious meat, domestic pigeon (*Columba livia*) has been widely raised commercially as a meat type poultry [1], and gradually became the fourth largest poultry in China. Unlike other poultry, as altrices, newly hatched pigeon squabs are initially fed with pigeon milk, which is secreted by the crop of their parents [2]. Composed of desquamated epithelial cells of their crop mucosa [3], crop milk is deficient in carbohydrate and mainly contains protein and lipids which maintain squab growth [4,5]. As squabs grow up, crop milk is gradually mixed with increasing quantities of grains derived from the parental diet and finally replaced by these grains completely [6], which results in the lipids of crop milk displaying significant quantitative changes in the feeding period. Besides being synthesized in the liver, almost all the lipids which accumulate in the body are derived from the diet [7]. Since lipid intake changes with age, as an atherosclerosis-susceptible breed [8,9,10], *Columba* squabs might have age-related alteration in lipid metabolism.

Given that serum lipid levels are good indicators of health status [11], and that inflammatory, innate immune processes and oxidative stress are regulated by lipids [12], we hypothesized that antioxidant status in pigeon squabs would also alter with age. In farm animals, oxidative stress might be concerned with conditions that were involved in animal production and the general welfare of individuals [13]. Besides impairing the feed efficiency, production performance, physiology, metabolism, and health of poultry, oxidative stress is one of the main factors limiting the quality and acceptability of poultry products [14]. However, research on antioxidant status in pigeon squabs as meat type poultry is limited.

Therefore, the objective of this study was to evaluate the age-related changes in the antioxidant status and lipid metabolism of pigeon squabs (*Columba livia*), by determining the BW, antioxidant indices, serum lipid levels, lipid metabolism-related enzyme activities, lipid metabolism-related gene expression, and liver morphology in squabs. 

## 2. Materials and Methods

All experimental protocols involving animals were approved by the Animal Care and Welfare Committee of Animal Science College and the Scientific Ethical Committee of Zhejiang University (No. ZJU2013105002) (Hangzhou, China). 

### 2.1. Experimental Birds and Conditions

A total of 200-pair parent White King pigeons in 60-week-old breeder flock were obtained from a commercial pigeon farm (Wenzhou, China). An artificial aviary equipped with a perch and a nest was provided for each pair. Parent pigeons were randomly allocated to ten replications, each of 20-pair pigeons. All parent pigeons were supplied with a cereal based diet. The ingredients and nutrient levels of cereal based diet for parent pigeons are listed in Table 1. The pigeons were given water ad libitum and were fed twice daily (7:00 a.m. and 3:00 p.m.) throughout the experiment. 

Each pair of parent pigeons laid two eggs in a nest. Eggs were picked out and transferred to an artificially incubator for an 18-day incubation (55 ± 2% relative humidity and 38.1 ± 0.1 °C). In the meantime, fake eggs were put into parents’ nests to meet parents’ brooding characteristics. On the day of hatching, 400 artificially hatched squabs with similar BW were selected, pair-matched and assigned into the nests of parent pigeons, replacing the fake eggs. Each parent pair adopted 2 artificially hatched squabs. As described by a previous study, pigeon squabs were fed with crop milk, which was secreted by parent pigeons in a mouth-to-mouth manner [1,2]. The crop milk mainly contained 52.68–58.47% crude protein and 18.94–32.77% crude fat (on a dry-matter basis), according to previous work in our lab [15]. The ambient temperature was 18 to 26 °C. The relative humidity was 60 to 70%, and the photoperiod was 12 L:12 D throughout the total experiment period.

### 2.2. Sample Collection

On the day of hatching (DOH), days 7 (D7), 14 (D14) and 21 (D21) post-hatch, ten squabs (one squab from each replication, half male and female) were selected randomly for sampling. The squabs from DOH were weighed and slaughtered within 2 h after hatch, but before feeding. The squabs from other days of age were fasted for 12 h before weighing and slaughter. Blood samples from the squabs were obtained from the brachial vein before killing squabs, and were immediately drawn into 10 mL Eppendorf tubes, respectively. After the blood samples clotting, the serum was centrifuged at 3000 *g* for 10 min. Pure serum was aspirated by pipette, and stored at −80 °C in 1.5 mL Eppendorf tubes. These selected squabs were all killed by cervical dislocation (squabs with BW over 250 g were sedated before cervical dislocation). Livers of the squabs were sampled and immediately frozen in liquid nitrogen, then stored at −80 °C for subsequent analyses.

### 2.3. Serum Lipid Level Analyses

Serum samples were thawed at 4 °C for lipid analyses. Total triglyceride (TG), total cholesterol (TC), free fatty acid (FFA), very low-density lipoprotein cholesterol (vLDL), low-density lipoprotein cholesterol (LDL), and high-density lipoprotein cholesterol (HDL) in serum were measured by an automatic biochemical analyzer (Hitachi 7600-020, Hitachi, Co., Tokyo, Japan), using commercially available test kits (Beijing Sino-uk Institute of Biological Technology, Beijing, China). All the index analyses were determined according to the instructions of the manufacturer.

### 2.4. Hepatic Antioxidant Index Analyses

The liver samples were thawed and chopped into small pieces on ice. The 10% (w/v) homogenates of the liver were prepared in 10 mM phosphate buffer (pH 7.4) and centrifuged at 10,000 *g* for 15 min at 4 °C. The supernatant of homogenates was collected and stored at −80 °C to conduct an antioxidant index measurement. The antioxidant indices of liver were analyzed by the same method in triplicate. Glutathione peroxidase (GPH-Px), catalase (CAT), total superoxide dismutase (SOD), and malondialdehyde (MDA) were determined using commercial kits (Nanjing Jiancheng Bioengineering Institute, Nanjing, China). The above indices were measured spectrophotometerically (UV-2000, Unico Instruments Co.Ltd., Shanghai, China). All the procedures were carried out according to the manufacturers’ instructions. The activities/concentration were expressed as units per milligram of protein. 

### 2.5. Lipid Metabolism-Related Enzyme Activity Measurement

The supernatant of homogenized liver samples was prepared for the measurement of lipid metabolism-related enzyme activities. The liver homogenates were made using the same methods mentioned in the hepatic antioxidant index analyses. The activities of hepatic lipase (HL) and lipoprotein lipase (LPL) were determined by absorbance changes at a wavelength of 550 nm with commercial kits (Nanjing Jiancheng Bioengineering Institute, Nanjing, China). The activities of hormone-sensitive lipase (HSL), fatty acid synthetase (FAS) and acetyl CoA carboxylase (ACC) were determined by absorbance changes at a wavelength of 450 nm with commercial kits (Nanjing Jiancheng Bioengineering Institute, Nanjing, China). Furthermore, the 3-hydroxy-3-methyl glutaryl coenzyme A reductase (HMGR) activity was determined by absorbance changes at a wavelength of 340 nm with commercial kits (Nanjing Jiancheng Bioengineering Institute, Nanjing, China). All the analyses were done according to the instructions of the manufacturer. These activities of lipid metabolism-related enzymes were expressed as units per gram/milligram of protein.

### 2.6. RNA Extraction and Quantitative PCR Analyses

Total RNA of pigeon liver was extracted using the TRIzol procedure (Invitrogen, Carlsbad, CA), according to the instructions of the manufacturer. The extracted RNA was quantified by the UV absorbance ratio at 260 and 280 nm. The RNA integrity was verified by native RNA electrophoresis on 1.0 % agarose gel. The complementary DNA was synthesized from 2 μg total RNA by M-MLV reverse transcriptase (Takara, Dalian, China) with oligo dT-adaptor primer at 42 °C for 60 min, following the protocol of the manufacturer. 

The abundance of mRNA was assayed on a StepOne Plus Real-Time PCR system (ABI 7500, Applied Biosystems, Foster City, CA). The specific primers uesd for endogenous reference gene (β-actin), FAS, ACC, acyl-CoA 1 (ACO), carnitine palmitoyltransferase 1 (CPT1), peroxisome proliferator activated receptor γ (PPARγ), and peroxisome proliferator activated receptor α (PPARα) are indicated in Table 2. The SYBR Green Realtime PCR Master Mix (Toyobo Co., Ltd., Osaka, Japan) was used for PCR reaction. The PCR program consisted of an initial DNA denaturation of 95 °C for 60 s, followed by 40 cycles of 95 °C for 15 s, and 60 °C for 60 s. The pooled samples determined the standard curve. The efficiency of the real-time PCR primers against all detected genes was calculated by standard curves. Each sample is in triplicate, excluding the template reference. The specificity of the amplification products was confirmed at the end of PCR by a melting curve analysis. The β-actin was considered as an appropriate endogenous reference. The average gene expression relative to the endogenous reference for each sample was calculated according to the 2^−ΔΔCt^ method [16]. The calibrator for each gene in experiments was the average ΔCt value of DOH.

### 2.7. Hepatic Histological Analyses

Hepatic histological analyses were conducted based on a previous study [17,18]. Approximately 0.5 cm^3^ liver samples were collected and fixed in 10% neutral-buffered formalin solution for subsequent histological analyses. Each liver sample was dehydrated, cleared, and embedded in paraffin. Serial sections (5 μm) of liver were placed on glass slides to be stained with hematoxylin and eosin. Moreover, frozen sections (5 μm) of liver were stained with Oil-Red-O for evaluation of hepatic lipid accumulation change. The Oil-Red-O staining area was determined using the Image-pro plus 6.0 (Media Cybernetics, Inc., Rockville, MD, USA).

### 2.8. Statistical Analyses

The data obtained from this experiment were subjected to a one-way analysis of variance in SPSS 24.0 (SPSS Inc., Chicago, IL, USA) for Windows. The differences between means were tested by Tukey’s multiple range test. The effect of age was determined using orthogonal polynomials for linear and quadratic effects. The level of significance was chosen at *p* < 0.05.

## 3. Results

### 3.1. Body Weight 

**Figure 1:**
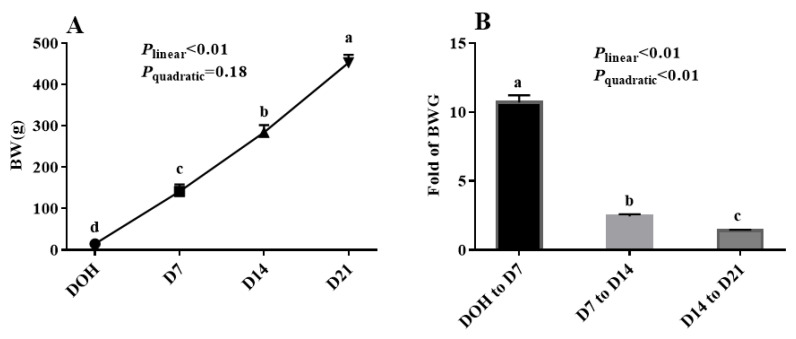
The age-related changes in BW and fold of BW gain (BWG) of domestic pigeon squabs. (**A**) BW, (**B**) BWG. DOH = day of hatch; D7 = day 7 post-hatch; D14 = day 14 post-hatch; D21 = day 21 post-hatch. Values are means ±SEM of 10 squabs, n = 10. ^a–d^ Means within the same day sharing no common superscripts differ significantly (Tukey test, *p* < 0.05).

The age-related changes in BW and fold of BW gain (BWG) of pigeon squabs are shown in Figure 1. The BW of squabs on D7 was 10.7 times that of squabs on DOH. The BW of squabs on D14 was 2.4 times that of squabs on D7. Moreover, the BW of squabs on D21 was 1.4 times that of squabs on D14. The BW of squabs increased linearly (*p* < 0.01) from DOH to D21. The fold of BWG in squabs responded to increasing age linearly (*p* < 0.01) and quadratically (*p* < 0.01), and the minimum response was observed in the phase from D14 to D21.

### 3.2. Serum Lipid Levels

The age-related changes in serum lipid levels of pigeon squabs are shown in Table 3. The levels of TG and FFA in squabs displayed linear (both *p* < 0.01) and quadratic (*p* = 0.01 and *p* < 0.01, respectively) trends, as the age increased with these maximum responses observed in D14. The LDL level responded to increasing age linearly (*p* < 0.01) and quadratically (*p* = 0.04), with the minimum observed in D14. The HDL level and HDL/LDL increased linearly (*p* < 0.01) as the age increased, whereas the vLDL level decreased linearly (*p* < 0.01) with age increasing. However, there was no significance (*p* > 0.05) in the TC level among four age groups.

### 3.3. Hepatic Antioxidant Capacity

**Figure 2:**
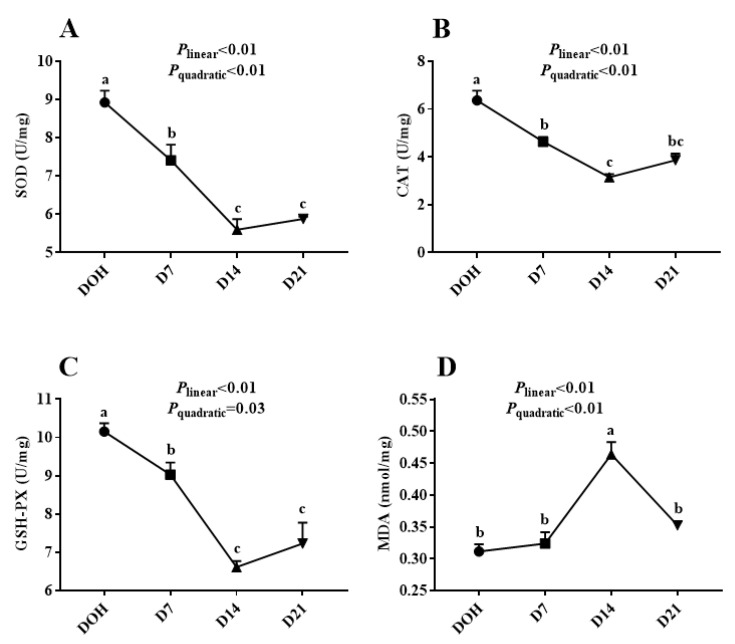
The age-related changes in hepatic antioxidant capacity of domestic pigeon squabs. DOH = day of hatch; D7 = day 7 post-hatch; D14 = day 14 post-hatch; D21 = day 21 post-hatch. (**A**) superoxide dismutase (SOD) activity, (**B**) catalase (CAT) activity, (**C**) glutathione peroxidase (GSH-Px) activity, (**D**) malondialdehyde (MDA) concentration. Values are means ±SEM of 10 squabs, n = 10. ^a–c^ Means within the same day sharing no common superscripts differ significantly (Tukey test, *p* < 0.05).

The age-related changes in the hepatic antioxidant capacity indices of pigeon squabs are shown in Figure 2. The activities of GSH-Px, CAT, SOD in squabs displayed linear (all *p* < 0.01) and quadratic (*p* = 0.03, *p* < 0.01 and *p* < 0.01, respectively) trends as the age increased, with these minimum responses observed in D14. However, the MDA concentration responded to increasing age linearly (*p* < 0.01) and quadratically (*p* < 0.01), with the maximum observed in D14.

### 3.4. Hepatic Lipid Metabolism-Related Enzyme Activities

**Figure 3:**
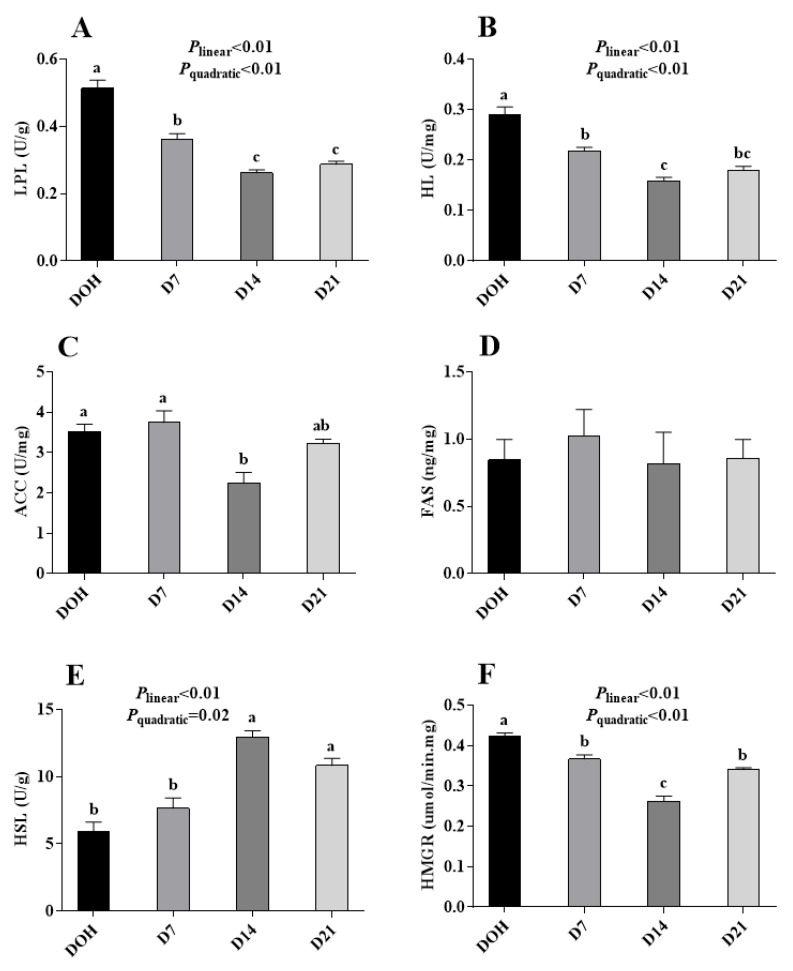
The age-related changes in activities of hepatic lipid metabolism-related enzymes of domestic pigeon squabs. (**A**) lipoprotein lipase (LPL) activity, (**B**) hepaticlipase (HL) activity, (**C**) acetyl CoA carboxylase (ACC) activity, (**D**) fatty acid synthetase (FAS) activity, (**E**) hormone-sensitive lipase (HSL) activity, (**F**) 3-hydroxy-3-methyl glutaryl coenzyme A reductase (HMGR) activity. DOH = day of hatch; D7 = day 7 post-hatch; D14 = day 14 post-hatch; D21 = day 21 post-hatch. Values are means ±SEM of 10 squabs, n = 10. ^a–c^ Means within the same day sharing no common superscripts differ significantly (Tukey test, *p* < 0.05).

The age-related changes in hepatic lipid metabolism-related enzyme activities of pigeon squabs are shown in Figure 3. The activities of LPL, HL, and HMGR in squabs responded to increasing age linearly (all *p* < 0.01) and quadratically (all *p* < 0.01), with these minimum responses observed in D14. The HSL activity displayed linear (*p* < 0.01) and quadratic (*p* = 0.02) trends as the age increased with the maximum observed in D14. Though the ACC activity in squabs on D14 was significantly decreased (*p* < 0.05) compared with that in squabs on DOH and D7, no significant (*p* > 0.05) linear or quadratic trend was observed. Besides, there was no significance (*p* > 0.05) in FAS activity among four age groups.

### 3.5. Hepatic Lipid Metabolism-Related Gene Expression

**Figure 4:**
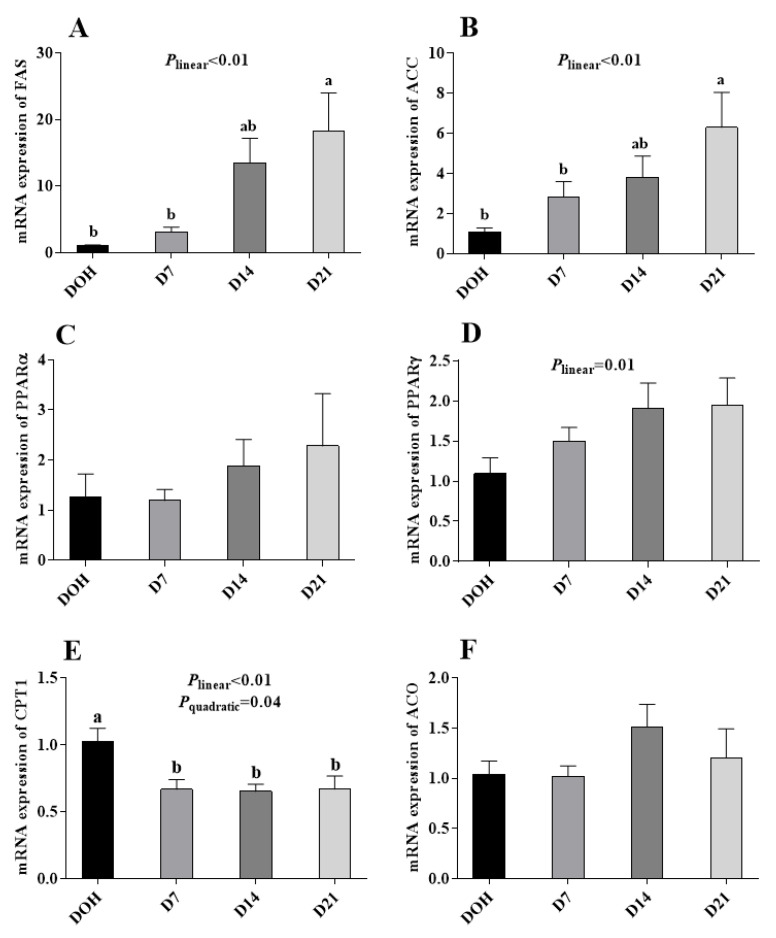
The age-related changes in the mRNA expression of hepatic lipid metabolism-related genes of domestic pigeon squabs. (**A**) mRNA expression of fatty acid synthase (FAS), (**B**) mRNA expression of acety l-CoA carboxylase (ACC), (**C**) mRNA expression of carnitine palmitoyltransferase 1 (CPT1), (**D**) mRNA expression of acyl-CoA (ACO), (**E**) mRNA expression of peroxisome proliferator activated receptor α (PPARα), (**F**) mRNA expression of peroxisome proliferator activated receptor γ (PPARγ). DOH = day of hatch; D7 = day 7 post-hatch; D14 = day 14 post-hatch; D21 = day 21 post-hatch. Values are means ±SEM of 10 squabs, n = 10. ^a,b^ Means within the same day sharing no common superscripts differ significantly (Tukey test, *p* < 0.05).

The age-related changes in the hepatic lipid metabolism-related gene expression of pigeon squabs are shown in Figure 4. The mRNA expression of CPT1 in squabs responded to increasing age linearly (*p* < 0.01) and quadratically (*p* = 0.04), with minimum response observed in D14, though no significance (*p* > 0.05) was observed among D7, D14 or D21. The mRNA expression of ACC and FAS increased linearly (*p* < 0.01). However, no significance (*p* > 0.05) in the mRNA expression of ACO, PPARα or PPARγ was observed among four age groups.

### 3.6. Hepatic histology and Lipid Accumulation

**Figure 5:**
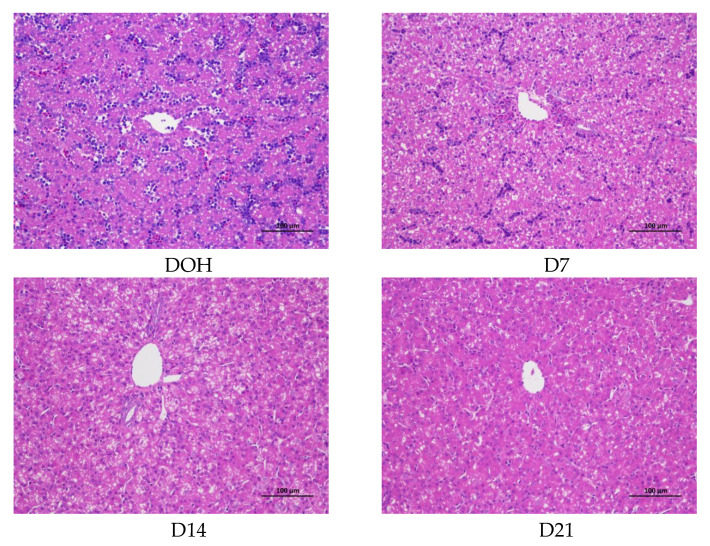
The age-related changes in hepatic morphology of domestic pigeon squabs. **DOH** = day of hatch; **D7** = day 7 post-hatch; **D14** = day 14 post-hatch; **D21** = day 21 post-hatch. Bar = 100 μm.

**Figure 6:**
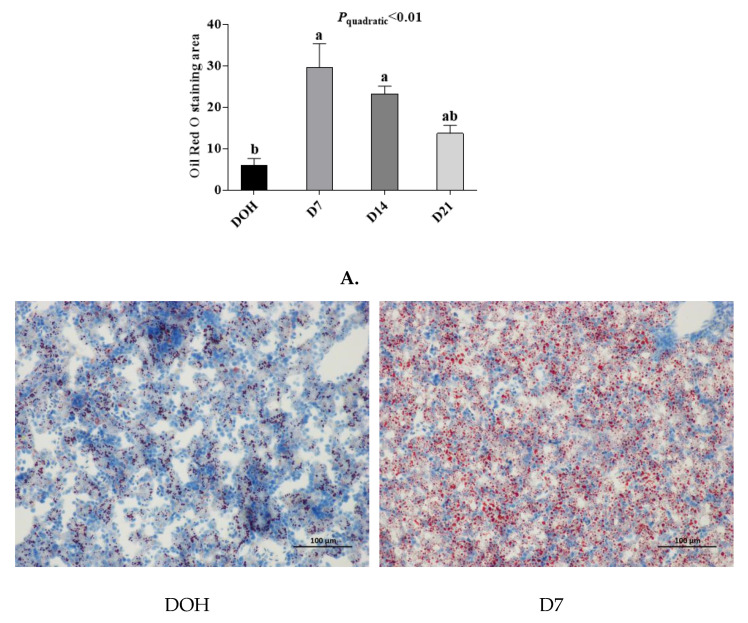

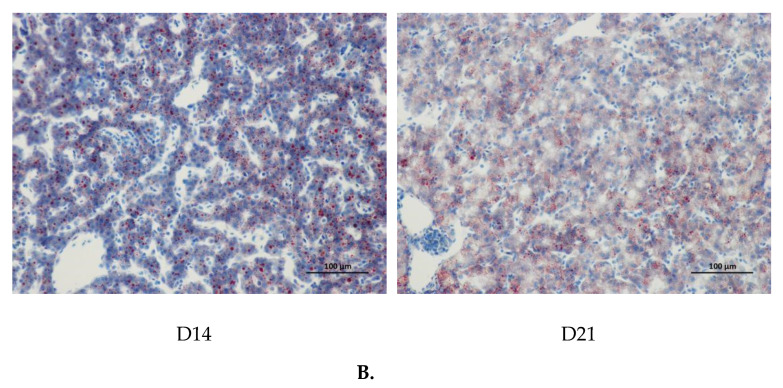
The age-related changes in hepatic lipid accumulation of domestic pigeon squabs. DOH = day of hatch; D7 = day 7 post-hatch; D14 = day 14 post-hatch; D21 = day 21 post-hatch. (**A**) Oil-Red-O staining area was measured using the Image-pro plus 6.0. Values are means ±SEM of 10 squabs, n = 10. ^a,b^ Means sharing no common superscripts differ significantly (Tukey test, *p* < 0.05). (**B**) Representative Oil-Red-O staining of livers in the four experimental groups. Bar = 100 μm.

The age-related changes in liver histology and lipid accumulation of pigeon squabs are shown in Figure 5 and Figure 6 respectively. Passing through the center of lobules, a central vein was radiating and arranging by hepatic cord or plate. Visually, in hematoxylin and eosin staining, clear hepatic plates were observed in squabs on DOH. However, visible hepatic plates decreased in squabs on D7, and the hepatic plates became unclear in squabs on D14 and D21. Besides, the Oil-Red-O staining area in squabs displayed a quadratic (*p* < 0.01) trend, as the age increased with the maximum response observed in D14.

## 4. Discussion

Domestic pigeons are kept for meat production and characterized by a superior growth rate [6]. The raising period of meat type pigeon is shorter than most other poultry species, resulting from that the inflection age of pigeons is earlier than ducks, chickens, geese, ostriches, turkeys, and Japanese quail [19]. In southern China, squabs reach the market weight of 400–500 g at 21 days of age. In the present study, squabs’ BW on D7 was nearly 10 times to that of DOH, and the BW on D14 was more than 20 times to that of DOH, which was similar with the experiment by Zhang et al. [20] and Xu et al. [1] The food that each young receives is of great importance for its growth [21]. The extraordinary relative growth rate in the phase from DOH to D7 might rely on the indispensable role of crop milk [18], which suggested that animal husbandry ought to provide suitable diet for parental pigeons to ensure maintain requirement and lactating requirement in this phase. After that phase, crop milk is mixed with increasing quantities of grains, such as corn and pea derived from the parent diet, and gradually replaced by these grains completely on approximately D14 [15,22]. The supply of crop milk should have an influence on the growth of the squabs [23]. Therefore, in the current study, the minimum fold of BWG in squabs was observed in the phase from D14 to D21.

In farm animals, oxidative stress might be concerned with the conditions that were involved in animal production and the general welfare of individuals [13]. Besides impairing the feed efficiency, production performance, physiology, metabolism, and health of poultry, oxidative stress is one of the main factors limiting the quality and acceptability of poultry products [14]. Oxidative damage occurs in birds because of an imbalance between the productions of reactive oxygen species and the animal’s defense mechanisms [24]. GSH-Px, CAT, and SOD are involved in non-enzymatic and enzymatic antioxidant defense mechanisms helping build strong antioxidant defenses [25]. On account of the critical roles played by SOD, CAT, and GSH-Px in a scavenging oxygen free radical, activities of these enzymes can be used as indicators of the antioxidant status [26]. In our current study, SOD, CAT, and GSH-Px activities displayed linear and quadratic trends as the age increased, with these minimum responses observed in D14, though no significant change was observed between D14 and D21. The results indicated that the antioxidant capacity of squabs was decreased from hatching, and became stable on D14. The inhibition of SOD, CAT, and GSH-Px activities contributes to the onset of many diseases [27]. Trichomonas, candidiasis, and crop indigestion are the most normal diseases in pigeon squabs. The addition of appropriate antioxidants might alleviate disease generation to some extent, by enhancing squabs’ antioxidant abilities. MDA is among the most studied products of polyunsaturated fatty acid peroxidation [28], and its lipid peroxidation is facilitated by reactive oxygen species [29]. The squabs on D14 in the current study displayed a highest MDA level, thus indicating that squabs in this phase went through more oxidative stress. The MDA content also serves as an indicator to determine serum lipid peroxidation levels. In the current study, the change trend of MDA content was consistent with that of serum FFA in squabs with age.

The triglyceride and cholesterol were transported as lipoprotein particles and free fatty acids from the origin to the target through blood circulation [30], which suggested serum TG, TC, HDL, LDL, vLDL and FFA concentrations were parameters to measure serum lipid levels [11]. In the current study, the higher TG levels were observed in squabs on D7 and D14. It is known that almost all the lipids which accumulate in the body are synthesized in the liver or derived from the diet [8]. As expected, more lipid droplets in hepatic morphology were observed in squabs of these two ages. The high serum TG level and lipid accumulation might be attributed to the crop milk, which is rich in lipids. There was no significant difference in the serum TC level among squabs of four ages. Nevertheless, the HDL/LDL increased linearly with age increasing since the serum HDL level in squabs increased linearly with squabs growing and serum LDL level was decreased on D7 and became statistically stable from D7 to D21. Besides, the vLDL level displayed a decreasing trend with age increasing. HDL is known as the “good” cholesterol, whereas vLDL and LDL are considered as “bad” types of cholesterol [31]. Therefore, the composition of serum TC became favorable for squabs with growing.

The balance of lipid metabolism is determined by lipogenesis and lipolysis. Unlike mammalian species, the liver is the main organ involved in lipogenesis in birds, as opposed to adipose tissue [32]. Enzymes such as FAS and ACC play important roles in the process of lipogenesis [11,33]. In the current study, no significance was observed, either in activity or mRNA expression, of ACC or FAS in liver between squabs on DOH and D7, suggesting that, in this phase, there was no significant change in fatty acid synthesis. Hence, the increase of serum TG and lipid accumulation on D7 might result from the decrease of lipolysis. Enzymes LPL, HL, and HSL are keys involved in lipid degradation [34,35,36]. Our results showed that the activities of LPL and HL in liver were decreased. Previous research indicated that HL has a major effect on the remodeling of LDL and is known to be regulated by several hormones, and possibly glucose and/or insulin [37]. LPL is a central enzyme in lipoprotein metabolism mediating lipolysis of triacylglycerols and phospholipids in circulating chylomicrons and vLDL [38]. Although it is known that LPL is a tissue-specific enzyme and is normally not made in the adult liver, it is expressed in the liver of newborn animals [39]. Adipose tissue LPL was regulated by serum glucose and insulin in normal weight human, but hepatic LPL responsiveness to insulin is not fully understood [40,41]. Notably, the decreasing trend of hepatic LPL activity in squabs with age was consistent with the description that LPL is expressed in the liver of newborn animals, and the hepatic LPL activity is extinguished during development [39]. The CPT1 is a key gene participating in the process of the β-oxidation of fatty acids [42]. In our study, the gene expression of CPT1 was down-regulated in squabs on D7. These results indicated that lipolysis in squabs on D7 might be decreased by inhibiting the hydrolysis of TG and the transportation of fatty acids through the inner membrane of mitochondria, via decreasing LPL and HL activities and CPT1 gene expression. In the phase from D7 to D14, the ACC activity was decreased, though unfortunately its gene expression had no significant difference. Ferraris and Diamond [43] also found that mRNA expression is not necessarily correlated with protein activity. So, we speculate that age might regulate ACC at a post-transcriptional level. Since the lipid accumulation in squabs on D14 was not significantly different from squabs on D7, the effects of decreasing ACC activity and increasing HSL activity might be equal to the effects of decreasing LPL and HL activities. In the phase from D14 to D21, neither enzyme activities nor the gene expression of lipid metabolism was observed, which was in line with the results of serum lipid levels and lipid accumulation, indicating that the lipid metabolism became stable from D14 to D21.

## 5. Conclusions

In conclusion, the phase from DOH to D14 was a growth spurt, especially in the first seven days. The antioxidant capacity of squabs had a continuous decline from DOH to D14. Besides, changes regarding lipid metabolism mainly occurred during the phase from DOH to D14. The first seven days showed mainly less lipid breakdown, while the second seven days displayed more complicated lipid metabolism. The results obtained from this study suggest that, in the pigeon industry, it is better to take nutritional manipulation in squabs before D14.

## Figures and Tables

**Table 1:** Ingredient compositions and nutrient levels of basal diets for parent pigeons ^1^ (on as-fed basis).

Items	Content
Ingredients of a whole-grain form feed (%)	
Corn	56.27
Pea	28.12
Wheat	5.63
Sorghum	5.63
Rapeseed oil	4.35
Total	100.00
Calculated nutrients ^2^ (%)	
Metabolizable energy (MJ/kg) ^3^	13.84
Crude protein	12.63
Ingredients of grit meal (%)	
Limestone	52.93
Shell meal	28.10
Yellow mud	14.05
Salt	1.41
Ferrous sulfate (monohydrate)	0.23
Premix ^4^	3.28
Total	100.00
Calculated nutrients (%)	
Calcium	27.79
Phosphorus	0.01
Sodium	0.03
Chlorine	0.59
Ferrum	0.30

^1^ All feeds were fed in a whole-grain form at 07:00 and 15:00 a day, and grit meal was offered to the birds on a continuous basis. ^2^ Nutrient values were calculated from tables of feed composition and nutritive values in China (twenty-eighth edition, 2017). ^3^ Metabolizable energy values determined in pigeons were calculated from those reported for chickens, in accordance with a previous study (Hullar et al. 1999), which observed that the metabolizable energy values of feed in pigeons did not differ significantly from those in chickens. ^4^ The premix provided the following per kg of diet: Vitamin A 5000 IU, Vitamin E 50 IU, Vitamin D_3_ 2000 IU, copper sulfate 15 mg, manganese sulfate 45 mg, zinc sulfate 90 mg.

**Table 2:** Primers used for quantitative real-time PCR analyses of gene expression in domestic pigeon squabs.

Genes ^1^	Accession Number ^2^	Forward (5′ ➔ 3′)	Reverse (5′ ➔ 3′)
β-actin	AB618546.1	CCCATCTACGAAGGCTACGC	CTTGATGTCACGCACAATTTC
FAS	XM_005515764.1	AAACTGAAGGCTGCTGATAAGT	CCTCCAATAAGGTGCGGTGAT
ACC	XM_013367232.1	CTCATGGTCTTCGCCAACTGGA	CACGATGTAGGCACCGAACTT
ACO	XM_005503118.2	GGCATTGAGGAGTGTCGGA	GCACAGTCACAGATGGAGCA
CPT1	XM_013369225.1	TCGTCTTGCCATGACTGGTG	GCTGTGGTGTCTGACTCGTT
PPARα	XM_021297326.1	AGAATAAGGAAGCCGAAGTTC	GGAGAAGCCAGGGATAGATTTG
PPARγ	XM_021288013.1	CCAGCGACATCGACCAGTT	GGTGATTTGTCTGTCGTCTTTCC

^1^ FAS = Fatty acid synthase; ACC = acety l -CoA carboxylase; ACO = acyl-CoA 1; CPT1 = carnitine palmitoyltransferase 1; PPARα = peroxisome proliferator activated receptor α; PPARγ = peroxisome proliferator activated receptor γ. ^2^ Accession number is from National Center for Biotechnology Information (https://www.ncbi.nlm.nih.gov/).

**Table 3:** The age-related changes in serum lipid levels of domestic pigeon squabs ^1^.

Items ^2^	TG (mmol/L)	TC (mmol/L)	HDL (mmol/L)	LDL (mmol/L)	HDL/LDL	vLDL (nmol/L)	FFA (nmol/L)
DOH	1.20^b^	8.57	2.04^c^	6.27^a^	0.35^b^	309.06^a^	242.99^c^
D7	3.25^a^	5.62	2.08^bc^	2.73^b^	0.70^ab^	249.15^ab^	383.48^b^
D14	3.81^a^	7.26	3.13^ab^	3.07^b^	1.07^a^	199.81^ab^	447.89^a^
D21	3.07^ab^	6.9	3.23^a^	3.09^b^	1.08^a^	161.63^b^	334.09^b^
P value	<0.01	0.14	<0.01	<0.01	0.02	0.02	<0.01
SEM	0.6	1.01	0.35	0.88	0.23	35.24	17.1
Contrast							
Linear	<0.01	0.17	<0.01	<0.01	<0.01	<0.01	<0.01
Quadratic	0.01	0.24	0.97	0.04	0.35	0.71	<0.01

^1^ DOH = day of hatch; D7 = day 7 post-hatch; D14 = day 14 post-hatch; D21 = day 21 post-hatch. ^2^ TG = triglyceride; TC = total cholesterol; HDL = high-density lipoprotein cholesterol; LDL = low-density lipoprotein cholesterol; HDL/LDL = the ratio of high-density lipoprotein cholesterol and low-density lipoprotein cholesterol; vLDL = very low-density lipoprotein cholesterol; FFA = free fatty acid. ^a-c^ Means (n = 10) within a row with no common superscripts differ significantly (*p* < 0.05).

## References

[1] Xu Q.Q., Zhang X.Y., Zou X.T., Dong X.Y. (2019). Effects of in ovo injection of L-histidine on hatch performance and post-hatch development in domestic pigeons (Columba livia). Poult. Sci..

[2] Horseman N.D., Buntin J.D. (1995). Regulation of pigeon cropmilk secretion and parental behaviors by prolactin. Annu. Rev. Nutr..

[3] Bharathi L., Shenoy K.B., Mojamdar M., Hegde S.N. (1993). Studies on the growth-stimulatory activity of pigeon milk--comparison and synergistic effects with serum. J. Comp. Physiol. B.

[4] Davies W.L. (1939). The composition of the crop milk of pigeons. Biochem. J..

[5] Gillespie M.J., Haring V.R., McColl K.A., Monaghan P., Donald J.A., Nicholas K.R. (2011). Histological and global gene expression analysis of the ‘lactating’ pigeon crop. BMC Genomics..

[6] Sales J., Janssens G.P.J. (2003). Nutrition of the domestic pigeon (Columba livia domestica). Worlds Poult. Sci. J..

[7] Peng M., Li L., Yu L., Ge C., Ma H. (2018). Effects of (−)-hydroxycitric acid on lipid droplet accumulation in chicken embryos. Anim. Sci. J..

[8] Chapman M.J. (1980). Animal lipoproteins: Chemistry, structure, and comparative aspects. J. Lipid. Res..

[9] Langelier M., Connelly P., Subbiah M.T.R. (1976). Plasma lipoprotein profile and composition in white carneau and show racer breeds of pigeons. Can. J. Biochem..

[10] Jensen P.F., Jensen G.L., Smith S.C. (1978). Serum lipoprotein profiles of young atherosclerosis-susceptible white carneau and atherosclerosis-resistant show racer pigeons. Comp. Biochem. Phys. B.

[11] Ma Z., Zhang J., Ma H., Dai B., Zheng L., Miao J., Zhang Y. (2014). The influence of dietary taurine and reduced housing density on hepatic functions in laying hens. Poult. Sci..

[12] Yu C.Y., Chen G.W., Cline D., Zhang H., Zong Y., Wang R. (2002). Mechanism by which fatty acids inhibit insulin activation of insulin receptor substrate-1 (IRS-1)-associated phosphatidylinositol 3-kinase activity in muscle. J. Biol. Chem..

[13] Lykkesfeldt J., Svendsen O. (2007). oxidants and antioxidant in diseases: Oxidative stress in farm animals. Vet. J..

[14] Jia R., Bao Y.H., Zhang Y., Ji C., Zhao L.H., Zhang J.Y., Gao C.Q., Ma Q.G. (2014). Effects of dietary α-lipoic acid, acetyl-L-carnitine, and sex on antioxidative ability, energy, and lipid metabolism in broilers. Poult. Sci..

[15] Zhang X.Y. (2018). Research on Early Development Regulation of Pigeon Squabs by Cationic Amino Acids. Ph.D. Thesis.

[16] Schmittgen T.D. (2001). Real-time quantitative PCR. Methods.

[17] Hünigen H., Mainzer K., Hirschberg R.M., Custodis P., Gemeinhardt O., Al-Masri S., Richardson K.C., Hafez H.M., Plendl J. (2016). Structure and age-dependent development of the turkey liver: A comparative study of a highly selected meat-type and a wild-type turkey line. Poult. Sci..

[18] Lynn E., Anne G., Anja B., Martin W., Andreas W., Arndt R., Kuhla A. (2018). Evaluation of two liver treatment strategies in a mouse model of niemann–pick-disease type c1. Int. J. Mol. Sci..

[19] Gao C.Q., Yang J.X., Chen M.X., Yan H.C., Wang X.Q. (2016). Growth curves and age-related changes in carcass characteristics, organs, serum parameters, and intestinal transporter gene, expression in domestic pigeon (columba livia). Poult. Sci..

[20] Zhang X.Y., Wan X.P., Miao L.P., Zou X.T., Dong X.Y. (2017). Effects of in ovo injection of l-arginine on hatchability, hatching time, early posthatch development, and carcass traits in domestic pigeons (Columba livia). J. Anim. Sci..

[21] Vandeputte-Poma J. (1980). Feeding, growth and metabolism of the pigeon, columba livia domestica: Duration and role of crop milk feeding. J. Comp. Physiol..

[22] Leash A.M., Liebman J., Taylor A., Limbert R. (1971). An analysis of the crop contents of white carneau pigeons (columba livia), days one through twenty-seven. Lab. Anim. Sci..

[23] Levi W.M. (1963). The Pigeon.

[24] Smet K., Raes K., Huyghebaert G., Haak L., Arnouts S., De Smet S. (2008). Lipid and protein oxidation of broiler meat as influenced by dietary natural antioxidant supplementation. Poult. Sci..

[25] Surai P.F., Kochish I.I. (2018). Nutritional modulation of the antioxidant capacities in poultry: The case of selenium. Poult. Sci..

[26] Bautista-Ortega J., Goeger D.E., Cherian G. (2009). Egg yolk omega-6 and omega-3 fatty acids modify tissue lipid components, antioxidant status, and ex vivo eicosanoid production in chick cardiac tissue. Poult. Sci..

[27] Baker J.S., Bailey D.M., Hullin D., Young I., Davies B. (2004). Metabolic implications of resistive force selection for oxidative stress and markers of muscle damage during 30 s of high-intensity exercise. Eur. J. Appl. Physiol..

[28] Skoie I.M., Dalen I., Omdal R., Jonsson G. (2019). Malondialdehyde and advanced oxidation protein products are not increased in psoriasis: A controlled study. Arch. Dermatol. Res..

[29] Zhou M., Zeng D., Ni X., Tu T., Yin Z., Pan K., Jing B. (2016). Effects of bacillus licheniformis on the growth performance and expression of lipid metabolism-related genes in broiler chickens challenged with clostridium perfringens-induced necrotic enteritis. Lipids. Health. Dis..

[30] Wnuk A., Mroczeksosnowska N.M., Łukasiewicz M., Batorska M., Niemiec J. (2013). Influence of the system of rearing on cholesterol level and its fraction in blood serum of slow-growing chickens. Ann. Warsaw Univ. Life Sci. SGGW Anim. Sci..

[31] Du X., Liu Y., Lu L., Wang W., Zeng T., Tian Y., Lu Y. (2016). Effects of dietary fats on egg quality and lipid parameters in serum and yolks of shan partridge duck. Poult. Sci..

[32] Badinga L., Selberg K., Dinges A., Corner C., Miles R. (2003). Dietary conjugated linoleic acid alters hepatic lipid content and fatty acid composition in broiler chickens. Poult. Sci..

[33] Joseph S.B., Laffitte B.A., Patel P.H., Watson M.A., Matsukuma K.E., Walczak R., Tontonoz P. (2002). Direct and indirect mechanisms for regulation of fatty acid synthase gene expression by liver x receptors. J. Biol. Chem..

[34] Niu Z.Y., Liu F.Z., Min Y.N., Li W.C. (2010). Effects of dietary dihydropyridine supplementation on growth performance and lipid metabolism of broiler chickens. Czech. J. Anim. Sci..

[35] Kersten S. (2014). Physiological regulation of lipoprotein lipase. Biochim. Biophys. Acta Mol. Cell. Biol. Lipids..

[36] Perret B., Mabile L., Martinez L., Tercé F., Barbaras R., Collet X. (2002). Hepatic lipase: Structure/function relationship, synthesis, and regulation. J. Lipid. Res..

[37] Schneider J.G., von Eynatten M., Schiekofer S., Nawroth P.P., Dugi K.A. (2005). Low plasma adiponectin levels are associated with increased hepatic lipase activity in Vivo. Diabetes Care.

[38] Lindberg A., Olivecrona G. (1995). Lipase evolution: Trout, Xenopus and chicken have lipoprotein lipase and apolipoprotein C-II-like activity but lack hepatic lipase-like activity. Biochim. Biophys. Acta, Lipids. Lipid. Metab..

[39] Liu W.M., Zhang J., Lu L.Z., Shi F.X., Niu D., Wang D.L. (2011). Effects of perilla extract on productive performance, serum values and hepatic expression of lipid-related genes in shaoxing ducks. Bri. Poult. Sci..

[40] Bloomgarden Z.T. (2005). Concepts of Insulin Resistance. Metab. Syndr. Relat. D.

[41] Taskinen M.R., Nikkilä E.A. (1981). Lipoprotein lipase of adipose tissue and skeletal muscle in human obesity: Response to glucose and to semistarvation. Metab. Clin. Exp..

[42] Merkel M., Eckel R.H., Goldberg I.J. (2002). Lipoprotein lipase. J. Lipid. Res..

[43] Ferraris R.P., Diamond J. (1997). Regulation of intestinal sugar transport. Physiol. Rev..

